# Classroom walkthroughs as an effective strategy for preschool improvement during the COVID-19 lockdowns: a Chinese model

**DOI:** 10.3389/fpsyg.2023.1154864

**Published:** 2023-06-02

**Authors:** Sha Xie, Hong Yin, Mengyun He, Hui Li

**Affiliations:** ^1^Faculty of Education, Shenzhen University, Shenzhen, Guangdong, China; ^2^Shanghai Institute of Early Childhood Education, Shanghai Normal University, Shanghai, China; ^3^Macquarie School of Education, Macquarie University, Sydney, NSW, Australia

**Keywords:** classroom walkthroughs, quality improvement, early childhood education, qualitative study, interview

## Abstract

Classroom walkthroughs are a widely used strategy for school improvement, varying over contexts and times. This study aims to explore the Chinese model of classroom walkthroughs in early childhood settings (ECS) during the COVID-19 lockdowns through a triangulated qualitative study. First, a group of ECS leaders (*N* = 15; *M*_year of teaching experience_ = 18.87, *SD* = 7.74, range = 6–33 years) and a group of teachers (*N* = 15; *M*_year of teaching experience_ = 8.40, *SD* = 3.96, range = 3–19 years) were interviewed in early 2022, and leaders’ observations notes were reviewed. The interview data were transcribed, recoded, and analyzed using an inductive approach, and the walkthrough documents were examined as a triangulation. Four themes and 13 subthemes emerged from the interview data: content, pedagogical skills, tasks, and challenges pertaining to classroom walk-throughs. Two major challenges against efficient classroom walkthroughs during the COVID-19 lockdowns were found: building community and feeding forward. Based on the results, a Chinese model of classroom walkthrough was proposed. Implications for quality improvement were also addressed.

## 1. Introduction

Classroom walkthroughs are a widely used strategy for quality improvement that requires both data-gathering and relationship-building continuously (Rouleau and Corner, 2020). This is because, as a school-changing supervisory practice, walkthroughs could facilitate teachers’ professional growth and, ultimately, (pre-)school improvement (Downey et al., 2004, 2009). There are varieties of classroom walkthroughs suited for different purposes and contexts, as local cultures and school systems have profoundly shaped them. Therefore, good walkthrough practices should be culturally and contextually appropriate to empower data collecting and relationship-building for school improvement. However, the existing studies have primarily focused on the walkthroughs in primary and secondary schools, leaving those in early childhood settings (ECS) underexplored (Muijs et al., 2004; Ressler et al., 2016; Perlman et al., 2020). Furthermore, the limited studies on ECS walkthroughs have focused on the American context (Perlman et al., 2020), neglecting the practices in other contexts, such as China, which has encountered the first outbreak of COVID-19 in the world. Since January 2020, Chinese schools and preschools have gone through repeated lockdowns, and the quality of teaching and learning has been deterred. Therefore, it is meaningful and timely to understand how Chinese preschools have practiced classroom walk-throughs during the lockdowns and thus to reflect on the lessons and experiences. To fill this research gap, the present study explored classroom walkthroughs through a triangulated qualitative study in southern China, aiming to provide a Chinese model of classroom walkthroughs.

### 1.1. Classroom walkthroughs as an effective approach for school improvement

Classroom walkthroughs are defined as “short, informal observations of classroom teachers and students by school administrators, coaches, mentors, peers, and others, followed by feedback, conversation, and/or action” (Stout et al., 2013, p. 1). Over the past years, it has been labeled with multiple names, such as “learning walks, instructional walks, walk-abouts, data walks, administrative walkthroughs, supervisory walkthroughs, reflective walkthroughs, and just walkthroughs” (Stout et al., 2013, p. 1); or “informal observations, pop-ins, walk-ins, or drop-ins” (Zepeda, 2013, p.18), with varied forms and processes. For example, Downey et al. (2004, 2009) developed a 3-min observation protocol to foster professional growth through reflective dialogue, while Colvin et al. (2009) conducted 10-min observations, provided feedback to teachers, and collaboratively developed action plans with teachers to improve instructional approaches in a high school. It is one form of instructional supervision and serves as a transformative tool to gather information to holistically support leaders, teachers, and student achievement and eventually guide improvement (Garza et al., 2016; Glickman et al., 2017).

Existing studies have preliminarily explored the factors related to classroom walkthroughs, such as directors’ characteristics and approaches that facilitated effective classroom walkthroughs and, eventually, quality improvement in early childhood settings (Rous, 2004; Liu et al., 2020; Perlman et al., 2020). Interestingly, directors’ characteristics showed no or even negative associations with supervision practices, and their working experiences were negatively related to quality scores using the Early Childhood Environment Rating Scale (Perlman et al., 2020). Participating in the walkthrough builds a sense of investment in the leaders (or supervisors) and grows into an effort to tailor teaching instruction and practice to help school improvement (Bole and Farizo, 2013; Moss and Brookhart, 2015, 2019). Therefore, supervisory approaches or pedagogical skills during classroom walkthroughs, instead of demographic characteristics, might lead to differences in quality improvement (Rous, 2004; Burns and Badiali, 2016, 2018; Glickman et al., 2017). Teacher mistrust, in contrast, can undermine walkthroughs (Romano, 2014; Khun-Inkeeree et al., 2019); thus, community and trust building are essential for the walkthrough to have an impact (Caruso and Fawcett, 2006; Glickman et al., 2017). However, most existing studies have focused on American contexts, leaving the classroom walkthroughs in other contexts underexplored (Rous, 2004; Garza et al., 2016; Khun-Inkeeree et al., 2019).

### 1.2. Classroom walkthroughs in Chinese early childhood settings

China’s early childhood education system consists of two levels: nurseries that provide childcare services to children aged 0–3 and kindergartens (preschools) that provide education and care services to children aged 3–6. This paper focuses on early childhood education for 3- to 6-year-olds, given its rapid development in the past decade. Early childhood education in China has gone through rapid change since the promulgation of the *Outline of National Plan for Medium- and Long-term Education Reform and Development* (2010–2020) (‘the Plan’) and the *State Council’s Several Opinions on the Current Development of Early Childhood Education* (‘the Opinions’) in 2010. The “3A” problem (accessibility, affordability, and accountability) since the 1990s was tackled by the Plan and the Opinions and ensuing national documents (Li et al., 2016; Xie and Li, 2020). Currently, the accessibility and affordability problems have been largely relieved by the establishment of Puhui (inclusive and inexpensive; 普惠) preschools, reflected by the gross enrollment rate rising from 50.9% in 2009 to 88.1% in 2021 (Ministry of Education, 2010, 2022a) and the restricted fees of the Puhui preschools (Xie and Li, 2020; Zhou et al., 2022). The problem of accountability, on the other hand, is an ongoing and continuous effort, as the Ministry of Education issued the *Guidelines on the Assessment of Preschool Care and Education Quality* in 2022 (‘the Guidelines’; Ministry of Education, 2022b), which marks the nation’s attention to improving quality in early childhood education.

Classroom walkthrough is an indispensable part of China’s teaching research system in early childhood education. It is also an essential work of preschool leadership in early childhood settings in China. First, there exists a well-established, multi-layered teaching research system through which the principal (director), deputy principal (director) of academic affairs, the teaching and research staff, and teachers work together to design, deliver, and revise their instructional practices to promote quality (Huang et al., 2010, 2014). Second, preschool principals are required by the *Professional Standard for Preschool Principals* to “establish a system of going into the classroom to guide care and education activities, using daily observations and planned to observe activities to understand and evaluate the care and education situation in a timely manner and give constructive feedback” (Ministry of Education, 2015). The principal (director), the deputy principal (director) of academic affairs, and the teaching and research staff, who constitute the management team or “leading team” of academic affairs, usually conduct classroom walkthroughs. However, with the rapid expansion of new preschools, many young directors are starting to lead the preschools without experience and training in effective classroom walkthroughs (Liu et al., 2020). Despite its prevalence in daily practice, classroom walkthrough is underexplored in the Chinese context, especially in early childhood education. Some Chinese studies have proposed different types of classroom walkthroughs for deputy directors of academic affairs (Ma and Li, 2014) and examined educators’ sense of the value of deputy directors’ practice of classroom practice (Liu et al., 2020). However, there lacks a comprehensive understanding of both the supervisors’ and supervisees’ views of classroom walkthrough and how it relates to quality improvement in Chinese early childhood settings, which is a remarkable research gap the current study intends to fill.

### 1.3. The current study

The COVID-19 pandemic has caused repeated lockdowns in China since 2020, Chinese preschools thus have to make more efforts to keep the quality of teaching and to learn under this difficult circumstance. Accordingly, Chinese principals and teachers must overcome all the challenges caused by the lockdowns to conduct classroom walk-throughs. This study aims to examine whether classroom walkthroughs serve the goal of quality improvement in early childhood education in China. The exploratory nature of this study allows illumination of the perceptions of preschool leaders and educators regarding the classroom walkthrough and its potential. In particular, the following research questions guided the current study:

1. What is the model of classroom walkthroughs commonly shared by Chinese early childhood leaders and educators?
2. What are the challenges to the classroom walkthroughs during the lockdowns?
3. Overall, does classroom walkthrough practice serve the goal of quality improvement in early childhood education in China?

## 2. Methods

### 2.1. Participants

The study took place in 2022 in the Shenzhen Special Economic Zone, one of China’s fastest-growing cities and the third-largest city in terms of GDP (Xie and Li, 2020). The only super-mega city neighboring Hong Kong, Shenzhen is the main entrance to Mainland China for those visitors from Hong Kong and overseas. However, since January 2020, Shenzhen has undergone three waves of city-wide lockdowns and multiple sub-district-wide quarantines to thoroughly implement the nationwide ‘Zero Case’ policy while receiving visitors from Hong Kong every day. Thus, Shenzhen has served as the frontline of the nation’s war against the COVID-19 pandemic. Accordingly, Shenzhen preschools and schools had to suspend or lock down repeatedly. Inevitably, this problematic situation has negatively affected the quality of teaching and learning in Shenzhen preschools, making it more challenging to conduct classroom walk-throughs as usual.

The research project was reviewed and approved by the first author’s University Human Research Ethics Committee, and the project was conducted in accordance with human subject guidelines. Eight public preschools were purposefully selected from Shenzhen to represent preschools of varied quality grading and in different developmental stages: some were public preschools from the start with varying years of establishment, and others were initially privately run but were transformed into publicly run. Preschool principals were first contacted and explained the purpose of the study. After their consent to participate in the study was given, we contacted and arranged one-on-one interviews with the supervisors (including principal/director, deputy principal/director of academic affairs, and the teaching and research staff; *N* = 15) and supervisees (teachers; *N* = 15). Fourteen of the 15 supervisors had bachelor’s degrees, and one had a master’s degree. Similarly, 14 out of the 15 supervisees had bachelor’s degrees, and one had an associate degree, showing no significant differences in their educational attainment. However, the supervisors had significantly more years of teaching experience than the supervisees (*M*_supervisor_ = 18.87, *SD* = 7.74, range = 6–33 years; *M*_supervisee_ = 8.40, *SD* = 3.96, range = 3–19 years; *t* = 4.67, *p* < 0.001).

### 2.2. Procedure

Each participant was contacted and scheduled for a semi-structured interview in a quiet room of the preschool they worked. Their consent to participate was obtained before the interview started and was recorded. The interview protocol was written in Mandarin Chinese, and all the interviews were conducted in Mandarin Chinese. During the interview, open-ended questions were asked with prompts to clarify participants’ responses or to elicit further discussion. Closing remark in which participants were given opportunities to ask questions and were thanked for their participation.

### 2.3. Measure

The semi-structured interview protocol was used. The following open-ended questions were relevant to the practices and perceptions of classroom walkthroughs in their preschool. Questions for the supervisors and supervisees were slightly different according to their roles (see Supplementary Appendix).

The interviews were audio-taped and transcribed, resulting in approximately 98 pages of single-spaced transcription for analyses.

### 2.4. Data analysis

The transcriptions were sent to the participants to verify that the transcriptions accurately represented their perceptions. The participants were encouraged to amend the transcripts when they felt that the transcripts did not communicate what they had intended to say or when they wanted to add additional information. Three participants returned transcripts with minor corrections on inaccurate transcription of individual words. After that, information identifying the participants was removed, and each transcript was assigned a code for further analysis.

The researchers were outsiders of the preschools in the current study and therefore an inductive approach was used to analyze the data and identify themes stemming from the data (Miles et al., 2019). Three coders coded the data. One coder held a Ph.D. in early childhood education, and two coders were master students in early childhood education and were trained to code classroom walkthroughs reliably. The first two coders examined the data line by line and independently coded the transcripts using NVivo version 11 (QSR International, Melbourne, Australia). Initial descriptive and process codes were identified in this first round of the coding phase (Miles et al., 2019). In the second round, connections and comparisons among the codes were examined to develop themes and subthemes (pattern coding; Miles et al., 2019, p. 86). The third coder read the transcript and identified the themes which were later discussed among all three coders. They discussed developing complete definitions and identifying areas of agreement and disagreement. During such a process, codes were revised or reduced, resulting in refined codes (Miles et al., 2019). In the third and final round, the two coders then re-read the transcripts to confirm the set of codes and themes; any coding differences were discussed until a complete agreement was reached. No new themes emerged, suggesting data saturation was achieved (Saunders et al., 2018). Links and relationships among the confirmed themes and subthemes were established. Additional coding was conducted to understand if the roles of the participants (i.e., supervisor or supervisee) offered unique insights.

To establish methodological triangulation and trustworthiness (Corbin and Strauss, 2014; Creswell, 2014), observation notes were also used to cross-check the interview analyses. The researcher collected all available observation notes with the leaders’ informed consent. Because supervisees (teachers) were usually observed and given written or oral suggestions by the supervisors, only observation notes by the supervisors were collected and analyzed in NVivo using the codes from the interview study to see if the findings were supported.

## 3. Results

### 3.1. Themes

**Figure 1:**
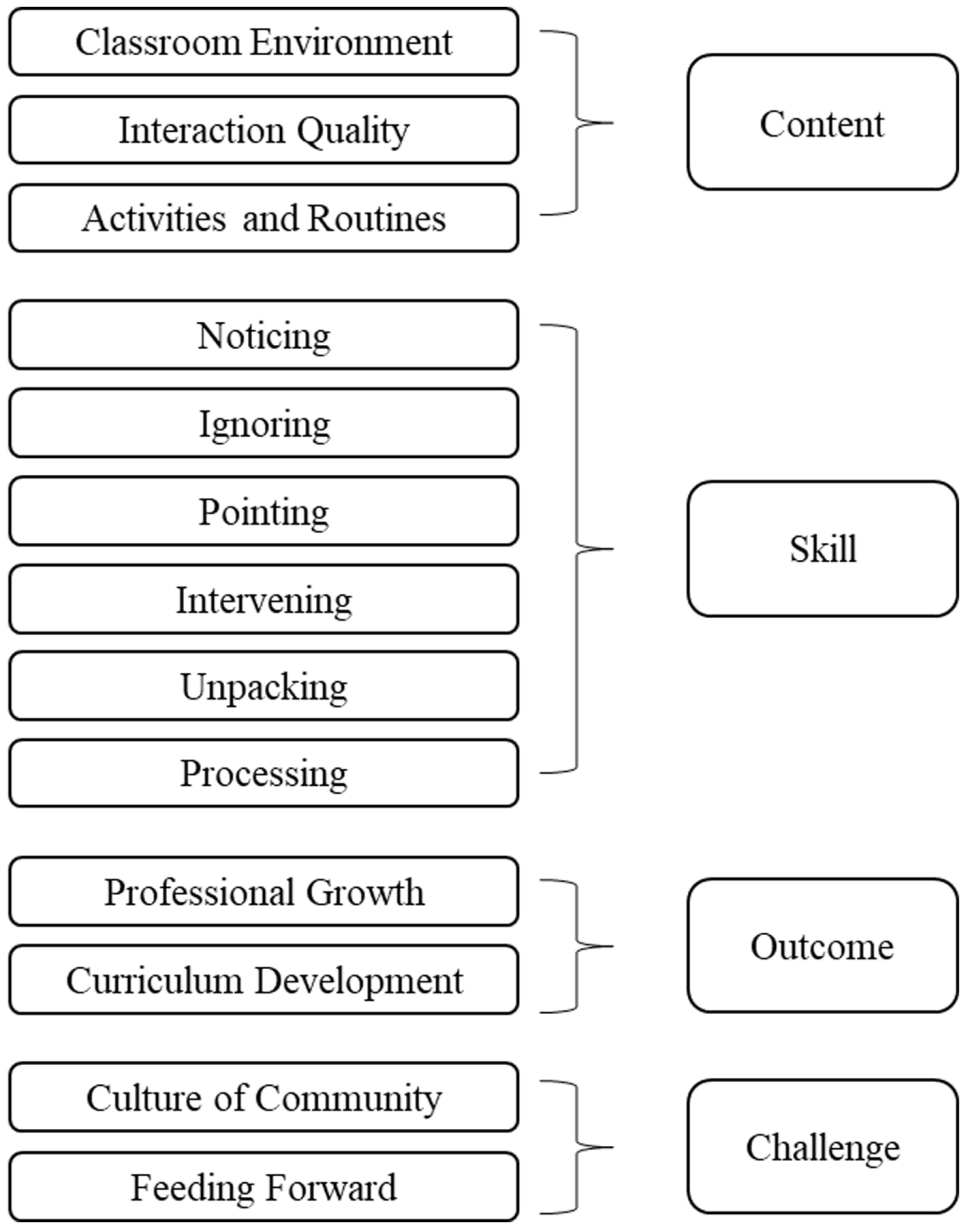
Themes and subthemes.

Figure 1 shows the four main themes and 12 subthemes that have emerged from the data analysis: content, pedagogical skill, outcome, and challenges pertaining to classroom walkthroughs. The participants described the content supervisors focused on, the interpersonal behaviors or strategies adopted during classroom walkthroughs, and the outcomes and challenges of classroom walkthroughs. Below we elaborate on these themes and ensuing subthemes and provide illustrative quotes as appropriate.

#### 3.1.1. Content of classroom walkthroughs

Three main contents were focused on during classroom walkthroughs: classroom environment, interactional quality, and activities and routines (Table 1). These three contents were considered crucial aspects of enhancing quality in preschool. During the brief walkthroughs, the supervisors would attend to these critical issues as indicators for supervision. It was interesting to find that the supervisors and supervisees expressed evenly of the contents, except for interaction quality, with twice as many supervisors mentioning interaction quality as the supervisees (46.67 and 20.00%, respectively). The observation notes supported that the supervisors generally focused on classroom environment, interaction quality, and activities and routines, with varied foci of attention during each walkthrough.

**Table 1:** Unique respondents who reported on the content of classroom walk-throughs.

Content of classroom walk-throughs	Supervisor *N* (%)	Supervisee *N* (%)	Quotations
Classroom environment	36.67%	30.00%	“Next, I will go to the learning corners to see whether children are interested and curious in exploring the materials, whether there is a wide range of provisions available, and whether the materials are age-appropriate.” (R6)
Interaction quality	46.67%	20.00%	“One more issue is teacher-child interaction. Are there too much or too few interactions? When a child encounters a challenge, does the teacher intervene at once, or does the teacher allow opportunities for exploration? I also check if the teachers are paying attention to details during children’s learning.” (R13)
			“I put a lot of attention in the details during teacher-teacher interactions, such as the communication between the teachers, including the tone of speech and attitude, as well as their body language.” (R11)
Activities and routines	46.67%	43.33%	“I would intentionally check on the progress of undergoing projects in each class. I will first look at the materials posted on the “project wall” and then ask the teachers to briefly walk me through the project.” (R5)

##### 3.1.1.1. Classroom environment

The participants described various issues in the environment that would be attended to during walkthroughs, with safety practices as “foremost important,” such as safety hazards that could result in severe injury or adequate supervision to protect children’s safety (e.g., supervision near areas of potential danger). When safety practices were ensured, materials provided in different learning corners were the following concern: noticing if the materials were appropriate and engaging. Finally, print and artwork displayed in the classroom were checked if they were related to the undergoing project or other classroom experiences (e.g., paints in spring colors when learning about seasons).

##### 3.1.1.2. Interaction quality

Two kinds of interaction quality were frequently mentioned as essential during classroom walkthroughs. The first was teacher-child interaction, which included whether the teacher supported children’s learning and critical thinking, whether a balance was maintained between the child’s need to explore independently and the teacher’s input into learning, and whether the teacher encouraged the development of mutual respect between children and adults.

The second was teacher–teacher interaction, where positive, warm, and supportive interaction models mutual respect for children. Furthermore, whether the responsibilities of each teacher in the room were clearly defined to ensure all children were supervised was also an important aspect during walkthroughs.

##### 3.1.1.3. Activities and routines

Participants mentioned that activities, routines, and transitions between activities were attended to during classroom walkthroughs, including the arrangement of the daily schedule, teacher’s talk during circle time, teachers’ instructions during group learning activities (projects), small-group learning activities and outdoor activities, personal care routines, as well as the transition between activities. It was worth noting that preschools might feature different focuses during classroom walkthroughs regarding the activities and routines. For example, participants from one preschool have highlighted areas featuring science learning as their focus, which was set as the target area of improvement for that term.

#### 3.1.2. Pedagogical skills of classroom walkthroughs

The conceptual framework for clinical pedagogy by Burns and Badiali (2018) was used, and the participants identified six pedagogical skills: noticing, pointing, ignoring, intervening, unpacking, and processing (Table 2). Because the skills focused on the supervisors, not the supervisees, there were more respondents from the supervisors than the supervisees who mentioned these six subthemes. The observation notes included descriptions of the content they observed and generally attached supervisors’ evaluations and suggestions on this content.

**Table 2:** Unique respondents who reported on the pedagogical skills of classroom walk-throughs.

Pedagogical skills of classroom walk-throughs	Supervisor *N* (%)	Supervisee *N* (%)	Quotations
Noticing	50.00%	33.33%	“During classroom walk-throughs, I would notice teachers or children have some issues that raised my attention. Usually, when I notice such an incident, I would first look around other classrooms to decide if it is a common or individual issue. If I find it a common issue, I will write it down and arrange a research seminar to discuss it.” (R7)
Ignoring	46.67%	23.33%	“But some teachers might be more sensitive, so that I would leave the classroom first. Because sometimes if I intervene immediately, the teacher might feel embarrassed and not lead to positive outcomes.” (R11)
Pointing	33.33%	43.33%	“Sometimes, when the supervisor noticed some minor or adjustable issues, she would talk to us face-to-face and give us some feedback on some rules of organizing activities or things to pay attention to.” (E3)
			“For some issues, I would send photos with some text descriptions to the teachers (supervisees). For example, I would describe what kind of incident was shown in the photo. I would try to use the simple description to point out the issue to the teacher.” (R6)
Intervening	33.33%	44.44%	“If it is a safety issue, I will immediately point it out or make a rapid adjustment.” (R6)
			“The supervisor will tell us on the spot how to use the materials to make it more fun. She will directly give us suggestions and also encourage us to make DIY learning materials and do more research.” (E11)
Unpacking	46.67%	20.00%	“For example, I will ask the teacher to share her feeling about the day or any reflections, and then I will pick up what the teacher said and provide my feedback. Or, I will directly describe the issue I noticed and ask the teacher what she was thinking at that moment. Based on the teacher’s response, I will share my thoughts.” (R4)
Processing	50.00%	36.67%	“If it is a common issue, I will mention it at the staff meeting and do some research with my teaching and research staff. For example, not long ago, I noticed during a classroom walk-through that the materials in the block areas were minimal in more than half of the classrooms. So I discussed with the teaching and research staff how to solve this problem.” (R10)

##### 3.1.2.1. Noticing

It was generally considered the first step during the walkthrough, as mentioned by participants of the regular or irregular behaviors noticed by the supervisors. Regular behaviors referred to whether teachers follow the expected schedule or protocol of teaching, and irregular behaviors referred to incidents that were distinguishable and marked for later discussion. Supervisors generally observed and identified these “critical issues,” and documented these incidents through physical markings, such as writing observation, taking pictures or videos or audio-recording, and mental marking, such as cognitive awareness of the incident. The supervisor then decided the next action step based on the incident’s nature, which led to other pedagogical skills discussed below.

##### 3.1.2.2. Ignoring

After noticing, supervisors may intend to ignore, with deliberate knowledge of something happening and deliberate choice of inaction. It was an elusive skill unless the supervisor disclosed that she noticed the incident and chose to ignore it. The reason for ignoring might be avoidance of interruption to the class or embarrassment to the supervisee. Thus, actions might be taken at late times.

##### 3.1.2.3. Pointing

It was an action after noticing, which drew another’s attention to a specific incident. Pointing included disclosure by sharing videos or pictures with others as a model of good practice and providing written or oral feedback to the supervisee. Both ways showed elements of directed attention by making the incident and the supervisors’ perspective visible.

##### 3.1.2.4. Intervening

It was an immediate action due to the incident noticed by the supervisor. Intervening was frequently used when supervisors noticed safety hazards to reduce the possibility of child injury. It was less frequently used to support the teacher’s instruction so that teachers may directly learn more about effective practice.

##### 3.1.2.5. Unpacking

It was a higher level of pointing by breaking down a complex critical incident into simpler components, with the intention of inducing reflective thinking in the supervisees. Unpacking may come in the form of telling, by describing the incident and extrapolating meaning out of the incident, or the form of questioning, by helping the supervisee to think and extrapolate.

##### 3.1.2.6. Processing

It was a reflection process that the supervisors and supervises understood the incident and decided upon the next step of action, usually in the form of preschool-wide professional learning activities or staff meetings. Incidents that were frequently noticed would be the target of discussion for professional learning activities, and both parties would derive potential actions together. Most importantly, these actions would be noticed by the supervisors in the next round to see if the issues were solved.

#### 3.1.3. Outcomes of classroom walkthroughs

There were two major outcomes of classroom walkthroughs identified by the participants, with teacher professional development frequently mentioned as one of the important functions of classroom walkthroughs and curriculum development as another function (Table 3).

**Table 3:** Unique respondents who reported on the tasks of classroom walk-throughs.

Tasks of classroom walk-throughs	Supervisor *N* (%)	Supervisee *N* (%)	Quotations
Professional growth	73.33%	93.33%	“Because during each classroom walk-through, the supervisors would provide some professional suggestions. For example, the supervisors provided advice on how to design diversified worksheets, scaffold children’s exploration, and diversify the materials in the learning corners. I would reflect on my practices and adjust my strategies each time after their feedback. This is very helpful to my professional growth.” (E4)
			“Sometimes, during classroom walk-throughs, I will stay in one classroom to observe and document. These are what we (as supervisors) need to do to understand teachers’ needs and support teachers’ professional growth, which is also a path for our own professional growth.” (R3)
Curriculum development	26.67%	93.33%	“For example, we were doing a project on tree house. Children were supposed to build a model, and they were very excited. When the supervisor came to our class, she gave me a very good idea. She said an excellent facility is near our preschool; we should share this resource with the children. She also advised me to buy certain things, to support children’s exploration using these materials in the classroom.” (E9)

##### 3.1.3.1. Professional growth

Participants, especially supervisees, frequently described that classroom walkthroughs constantly reminded teachers to do their best and sometimes noticed some inappropriate practices that teachers themselves did not realize, indicating the “normative” function of supervision that intended to ensure quality. Seven participants also described that the objective feedback, as well as timely assistance, supported teachers’ practices, indicating the “restorative” function of supervision. Finally, 21 participants responded that classroom walkthroughs provided opportunities for self-reflection on their actions which helped develop their strategies and skills, indicating the “formative” function of supervision.

Apart from teachers’ professional development, participants, especially supervisors, mentioned their professional development of themselves. The supervisors described the reflections on their own behaviors during classroom walkthroughs and how they adjusted their supervision skills to help teachers grow. Two of them have also expressed the concern of lack of experience in conducting classroom walkthroughs and instructional supervision, which echoed one supervisee who expressed her expectation upon the supervisors to hold certain competencies before conducting supervision. Four of the observation notes included supervisors’ own reflections on the content, such as question marks on the comment, indicating that the supervisors themselves were going through reflection toward professional development.

##### 3.1.3.2. Curriculum development

Another task of classroom walkthroughs is to support curriculum development. Supervisors would provide ideas to help supervisees design materials and deliver learning activities that can better support children’s learning.

#### 3.1.4. Lockdown-associated challenges to classroom walkthroughs

Aside from the contents, behaviors, and tasks of classroom walkthroughs, the participants also expressed the challenges of conducting efficient and productive classroom walkthroughs during the COVID-19 lockdowns (Table 4).

**Table 4:** Unique respondents who reported on the challenges of classroom walk-throughs.

Challenges of classroom walk-throughs	Supervisor *N* (%)	Supervisee *N* (%)	Quotations
Building culture of community	66.67%	73.33%	“Honestly speaking, I do not want to be observed. If there is no classroom walk-through, I will not feel the pressure from outside, so that I could be myself and flexibly arrange the learning activities.” (E8) “The supervisors do not come to nitpick; they come to provide professional suggestions. When I realize that, I become less nervous and want to receive suggestions on professional growth during classroom walk-throughs. Furthermore, I want tailor-made walk-throughs. For example, when I think there are some problems in the transition, I can invite the supervisors to come and give us some suggestions. Though this is a very romantic way, I still think this can build teacher agency.” (E14)
Feeding forward	26.67%	0%	“As deputy principal of academic affairs, I have to supervise 12 classrooms, which adds up to more than 30 teachers. Therefore, after providing feedback during the walk-through, I have no idea if the teacher solved the problem. It is a big challenge for me to follow up with each of them.” (R6)

##### 3.1.4.1. Building culture of community

More than half of the participants expressed that supervisees were usually nervous and even overwhelmed when supervisors walked through the classroom, with very few describing themselves as being “business as usual.” Stemming from this nerve caused by the COVID-19 lockdowns, there was a split between whether the supervisees expected to be observed or not, with five of supervisees expressed their expectations that the supervisors could provide new insights during classroom walkthroughs while others did not wish to be observed unnoticed but welcomed planned or invited observations. Finally, it was highlighted that authentic and collegial relationship with supervisees based on respect, compassion, and the common goal of improving children learning was crucial to maximizing the benefit of classroom walkthroughs. Thus, supervisors should strive to build a culture of community in achieving that goal, especially during difficult times.

##### 3.1.4.2. Feeding forward

During the classroom walkthrough, feedback was usually provided on the spot. However, three supervisors expressed the difficulty in following up with the issues noticed during the walkthrough, thus limiting the potential of classroom walkthroughs to “feed forward” the teaching and learning activities. It was interesting to find that none of the supervisees mentioned the problem of feeding forward, highlighting that the supervisees might not be aware of such a problem.

### 3.2. Model of classroom walkthrough in early childhood education

**Figure 2:**
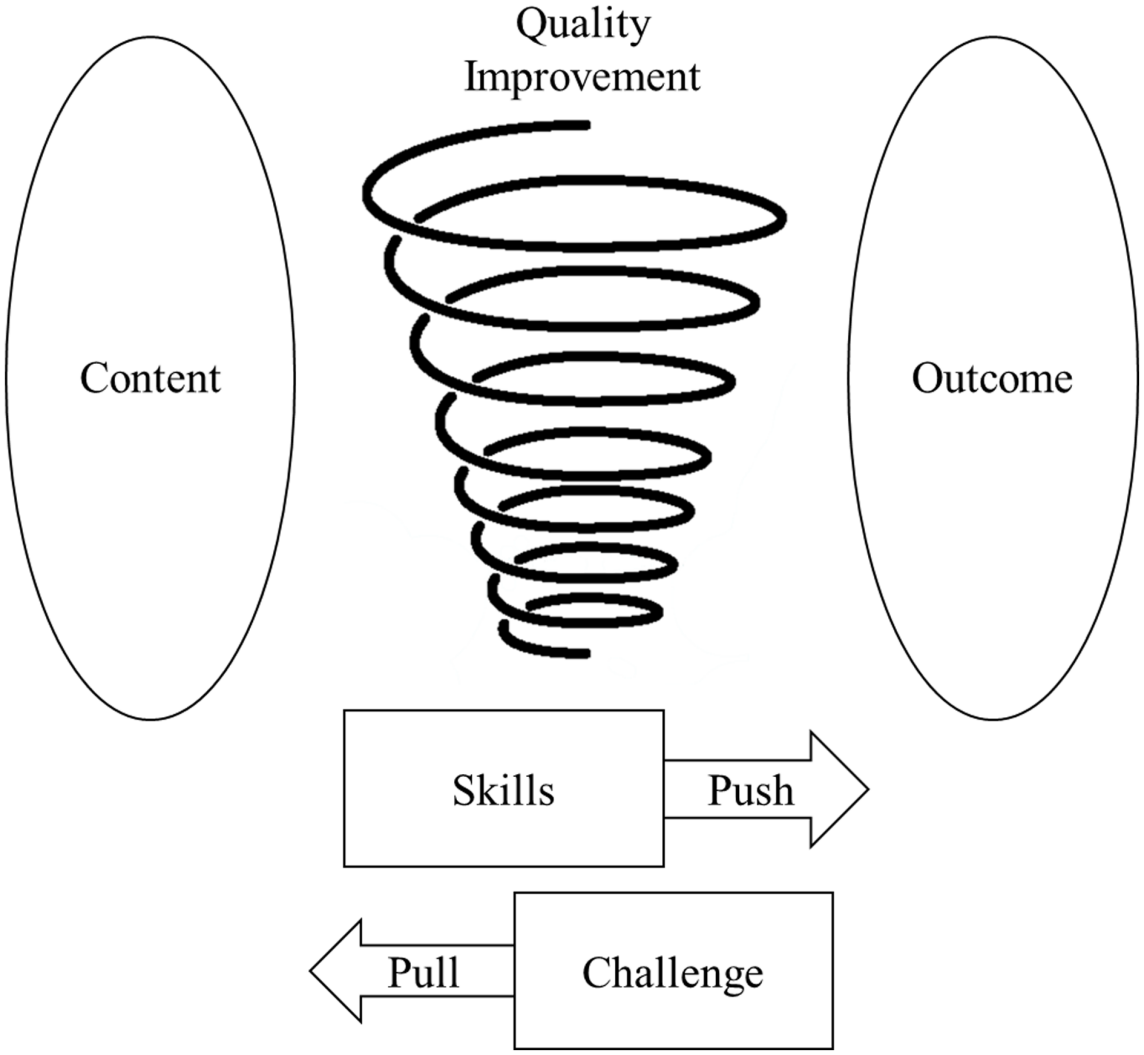
Model of classroom walk-through in early childhood education.

After a review of the themes and codes, a model of classroom walkthrough in early childhood education is proposed (see Figure 2). In this model, the *content* serves as the “focus” or the “tangibles” during classroom walkthroughs, through which supervisors become familiar with the teaching patterns and decisions that teachers make on a daily basis. The *outcome* serves as the “purpose” of classroom walkthroughs, which is also the purpose of instructional supervision: to promote both the supervisors’ and supervisees’ professional development and curriculum development. Down in the figure, there were two forces that were driving the classroom walkthroughs: a push force defined as pedagogical skills for classroom walk-throughs and a pull force defined as challenges confronting classroom walk-throughs. A better set of skills displayed by the supervisors would drive toward better outcomes, such as building reflective thinking in both supervisors and supervisees, an indicator of professional growth. On the contrary, the challenges associated with lockdowns hindered the endeavor toward better outcomes. Finally, a developmental perspective was adopted in this model in which the spiral course indicated that classroom walk-throughs were not a one-time effort. Instead, they should be brief, regular, and sustained over a long time, struggling between the push and pull forces in order to obtain quality improvement over time.

## 4. Discussion

Classroom walkthrough has become a prominent tool for instructional leadership practice that may involve leaders and educators in a collaborative process to observe, analyze, and evaluate the appropriateness of instructional practice (Caruso and Fawcett, 2006; Glickman et al., 2017) and eventually promote effective practice and achieve quality improvement (Ovando and Ramirez, 2007; Garza et al., 2016). This exploratory study revealed the content, outcome, skills, and challenges pertaining to classroom walkthroughs in China during the COVID-19 lockdowns and proposed a model of classroom walkthrough to depict how classroom walkthrough serves as an effective approach toward quality improvement in preschools.

### 4.1. Chinese model of classroom walkthroughs

First, this study found that the content of the classroom walkthroughs included classroom environment, interaction quality, as well as activities and routines in the classroom. Comparing them with the standardized quality assessment tools such as ECERS, Classroom Assessment Scoring System (CLASS), and Individualized Classroom Assessment Scoring System (inCLASS) and the national quality assessment guidelines revealed that the commonly used indicators in the assessment tools can be observed during classroom walkthroughs, providing evidence that walkthrough can be a competent observational tool for improving instructional practice (Zepeda, 2014). However, there are scant studies that examined the quality improvement of early childhood education programs over time (Kuger et al., 2016), and among the limited evidence, one study found that there was a substantial quality improvement in CLASS across the state of Louisiana, U.S.A. and that this improvement was driven by within-program changes (Bassok et al., 2021). The driving force of such improvement was substantial federal, state, and local investments, which are external efforts. On the contrary, supervision leadership, including classroom walkthroughs, are internal efforts toward quality improvement, which are more sustainable and pervasive (Downey et al., 2004, 2009; Glickman et al., 2017).

Second, this study found the outcome of classroom walkthroughs, including professional growth and curriculum development. In particular, the impact of classroom walkthroughs on the professional growth of educators corroborated with existing evidence that found leaders’ supervisory practices were related differently to educators’ performance, competency of teaching, and teaching efficacy (Darishah et al., 2017; Khun-Inkeeree et al., 2019, 2020; Hoque et al., 2020). However, the relationship between classroom walkthroughs and the professional growth of the center leaders was less explored in the literature, calling for more support to center leaders, such as observational tools, in their efforts to improve ECEC quality. The findings also revealed the role of classroom walkthroughs in curriculum development, with educators expressing their need for sustained support from the leaders in developing the curriculum, highlighting how ECE leaders provide appropriate and sustainable curriculum practices (Yang, 2019). It is worth noting that too much top-down monitoring limits educators’ autonomy in curriculum decision-making (Li and Chen, 2017; Yang, 2019), and therefore the findings also highlighted educators’ preference for leaders to “answer” their specific needs rather than guidance in general.

Therefore, this study proposed a model of classroom walkthrough in Chinese preschools, putting important factors into a unified model. This model used a developmental perspective, recognizing the push (pedagogical skills) and pull (challenges) forces that drive quality improvement in the long term. The current findings suggested that classroom walkthrough can serve as an effective approach toward sustainable quality improvement in early childhood education (Caruso and Fawcett, 2006; Stout et al., 2013; Moss and Brookhart, 2019), though much effort and training is needed to reach the goal of quality education for our young children.

### 4.2. Challenges and pedagogical skills

First, this study revealed two major challenges against efficient classroom walkthroughs during the COVID-19 lockdowns. The first challenge, building community in the center, corroborates with educators’ prevailing mixed attitudes toward such supervision practice in existing studies (Darishah et al., 2017; Khun-Inkeeree et al., 2019; Hoque et al., 2020). Teachers generally agree that classroom walkthroughs would benefit their professional development and curriculum development as a whole, but interpersonal skills were the barriers, as was found in a study in Malaysia (Khun-Inkeeree et al., 2019). More studies are needed to explore the effective walkthrough process in promoting a collaborative learning culture (Moss and Brookhart, 2015, 2019; Glickman et al., 2017). The second challenge, feeding forward, though less explored in the existing literature, can be instrumental in helping both leaders and educators better understand the need for evidence-based change and improvement in instructional practices and deserve further research (Stout et al., 2013; Moss and Brookhart, 2019).

According, this study also explored the pedagogical skills pertaining to classroom walkthroughs, using the framework by Burns and Badiali (2016, 2018). The findings found that the six pedagogical skills were important for enhancing school effectiveness, which is consistent with the existing evidence that leaders’ supervision practices contribute to the quality of ECE programs (Bloom and Sheerer, 1992; Vu et al., 2008; Bloom and Abel, 2015). A more recent study in Australia, however, found a lack of association between director’s supervision practices and classroom quality indicated by ECERS-E and CLASS (Perlman et al., 2020), calling for more research to explore the impact of supervisory practice. Furthermore, the findings indicated that the process of learning how to supervise well could be challenging for the supervisors (leaders) since the lack of in-service training for supervisory skills, especially those pertaining to classroom walkthroughs (Burns and Badiali, 2016; Perlman et al., 2020).

## 5. Limitations and conclusion

The limitations of the current study are worth mentioning. First, the current study only utilized interview and document analysis to understand ECE leaders’ and educators’ perspectives on classroom walkthroughs. Future studies that include questionnaires and classroom observation would substantiate and triangulate the findings from interviews. Second, the current study was conducted in one city in China, with might not be representative of all the ECE settings, especially those in less developed parts of the country. The future study shall compare and investigate whether there were differences in the content, skills, outcome, and challenges of the classroom walkthrough in centers from different socioeconomic backgrounds. Last but not least, this study was conducted during the lockdowns; without the data collected before the outbreak of the COVID-19 pandemic, it is impossible to compare the differences in classroom walkthroughs between the lockdown and usual times.

Despite the limitations, the current study explored ECE leaders’ and teachers’ perspectives on classroom walkthroughs, investigated the content, skills, outcome, and challenges pertaining to classroom walkthroughs, and proposed a Chinese model of classroom walkthrough in early childhood education from a developmental perspective. The findings implied that a classroom walkthrough is an effective approach in improving quality even during the lockdowns, yet more in-service training for ECE leaders is needed to equip ECE leaders with appropriate pedagogical skills and to build a sense of community within the center to reach the goal of sustainable quality improvement. Furthermore, the findings of the current study provided implications for its use in other contexts, given the generally shared understanding in what is quality in early childhood education (Siraj-Blatchford and Wong, 1999) and how classroom walkthrough supports quality improvement in daily practices (Caruso and Fawcett, 2006).

## Data availability statement

The raw data supporting the conclusions of this article will be made available by the authors, without undue reservation.

## Ethics statement

The studies involving human participants were reviewed and approved by Shenzhen University. The patients/participants provided their written informed consent to participate in this study.

## Author contributions

SX and HL: conceptualization. SX: methodology, data curation, writing – original draft preparation, and supervision. HY and MH: formal analysis and project administration. HL: writing – review and editing. All authors have read and approved the final manuscript.

## Funding

This work was supported by the Guangdong Planning Office of Philosophy and Social Science, China (grant number GD21YJY09), Shenzhen Planning Office of Philosophy and Social Science (grant number SZ2022B031), and Shenzhen Education Science Planning Project, China (grant number zdzz21002).

## Conflict of interest

The authors declare that the research was conducted in the absence of any commercial or financial relationships that could be construed as a potential conflict of interest.

## Publisher’s note

All claims expressed in this article are solely those of the authors and do not necessarily represent those of their affiliated organizations, or those of the publisher, the editors and the reviewers. Any product that may be evaluated in this article, or claim that may be made by its manufacturer, is not guaranteed or endorsed by the publisher.

## Supplementary material

The Supplementary material for this article can be found online at:

https://www.frontiersin.org/articles/10.3389/fpsyg.2023.1154864/full#supplementary-material
