# Supplementary material for: Classroom walkthroughs as an effective strategy for preschool improvement during the COVID-19 lockdowns: a Chinese model
Source: Front Psychol. 2023 Jun 2;14:1154864. doi: 10.3389/fpsyg.2023.1154864 (PMC10272574; doi:10.3389/fpsyg.2023.1154864)
Supplement: Supplementary file 1 [file Data_Sheet_1.docx]

Supplementary Material

Classroom Walkthroughs as an Effective Strategy for Preschool Improvement During the COVID-19 Lockdowns: A Chinese Model

# Interview Protocol for Supervisors (Leaders)

(1) How often do you walk into the classroom this semester?

(2) What do you do during walkthroughs?

(3) What are the purposes of classroom walkthroughs?

(4) What is your perception of the current practice of classroom walkthroughs?

# Interview Protocol for Supervisees (Teachers)

(1) How often do your supervisors walk into the classroom this semester?

(2) What do your supervisors do during walkthroughs?

(3) What are the purposes of classroom walkthroughs?

(4) What is your perception of the current practice of classroom walkthroughs?
